# Case report: Obturator hernia: Diagnosis and surgical treatment

**DOI:** 10.3389/fsurg.2023.1159246

**Published:** 2023-04-25

**Authors:** Han Li, Xuefeng Cao, Lingqun Kong

**Affiliations:** ^1^ Department of Hepatobiliary Surgery, Zibo Central Hospital, Zibo, China; ^2^ Binzhou Medical University, Binzhou, China; ^3^ Department of Hepatobiliary Surgery, Binzhou Medical University Hospital, Binzhou, China

**Keywords:** obturator hernia, old woman, diagnose, treatment, case report

## Abstract

**Background:**

Obturator hernia (OH) is a rare external abdominal hernia, accounting for only 0.07%–1% of all hernia cases. Because the female pelvis is wider and there is less preperitoneal adipose tissue, the obturator canal is larger, which can lead to herniation of abdominal contents when abdominal pressure increases in elderly women with thin body. The clinical symptoms of patients with obturator hernia included abdominal pain, nausea, vomiting, etc., and the mass in the inguinal region could not be touched. The positive Howship-Romberg sign is a specific sign of OH. CT is the first choice for the diagnosis of obturator hernia. Since intestinal incarceration in OH patients is prone to lead to intestinal necrosis, emergency surgical treatment is often required. However, due to the lack of specificity of its clinical manifestations, the misdiagnosis rate is high, which often leads to the delay of diagnosis and treatment.

**Methods:**

We report the case of an 86-year-old woman with a thin body and a history of multiple deliveries. The patient presented with abdominal pain, bloating, and constipation for 5 days. Physical examination revealed a positive Howship-Romberg sign on the right side, and CT examination suggested intestinal obstruction. Therefore, an urgent exploratory laparotomy was performed.

**Results:**

After opening the abdominal cavity we found that the wall of the ileum was embedded in the right obturator, and the proximal bowel was significantly dilated. We restored the embedded bowel wall to its original position, resected the necrotic bowel and performed an end-to-end anastomosis of the small intestine. The right hernia orifice was sutured, and OH was diagnosed during the operation.

**Conclusion:**

This article summarizes the diagnosis and treatment of OH by sharing this case, so as to provide a more detailed plan for early diagnosis and treatment of OH.

## 1. Introduction

Obturator hernia (OH) is a rare external abdominal hernia formed by protrusion of the abdominal visceral organs or extraperitoneal fat through the obturator of the hip bone to the femoral triangle. It was first reported by de Ronsil in 1724. OH has a relatively low incidence, accounting for only 0.07%–1% of all hernias. It is most common in thin elderly women. The incidence rate in women is 6–9 times higher than in men (1–3). A patient with OH who presented to our hospital is described below.

## 2. Case presentation

### 2.1. Patient information

**Figure 1:**
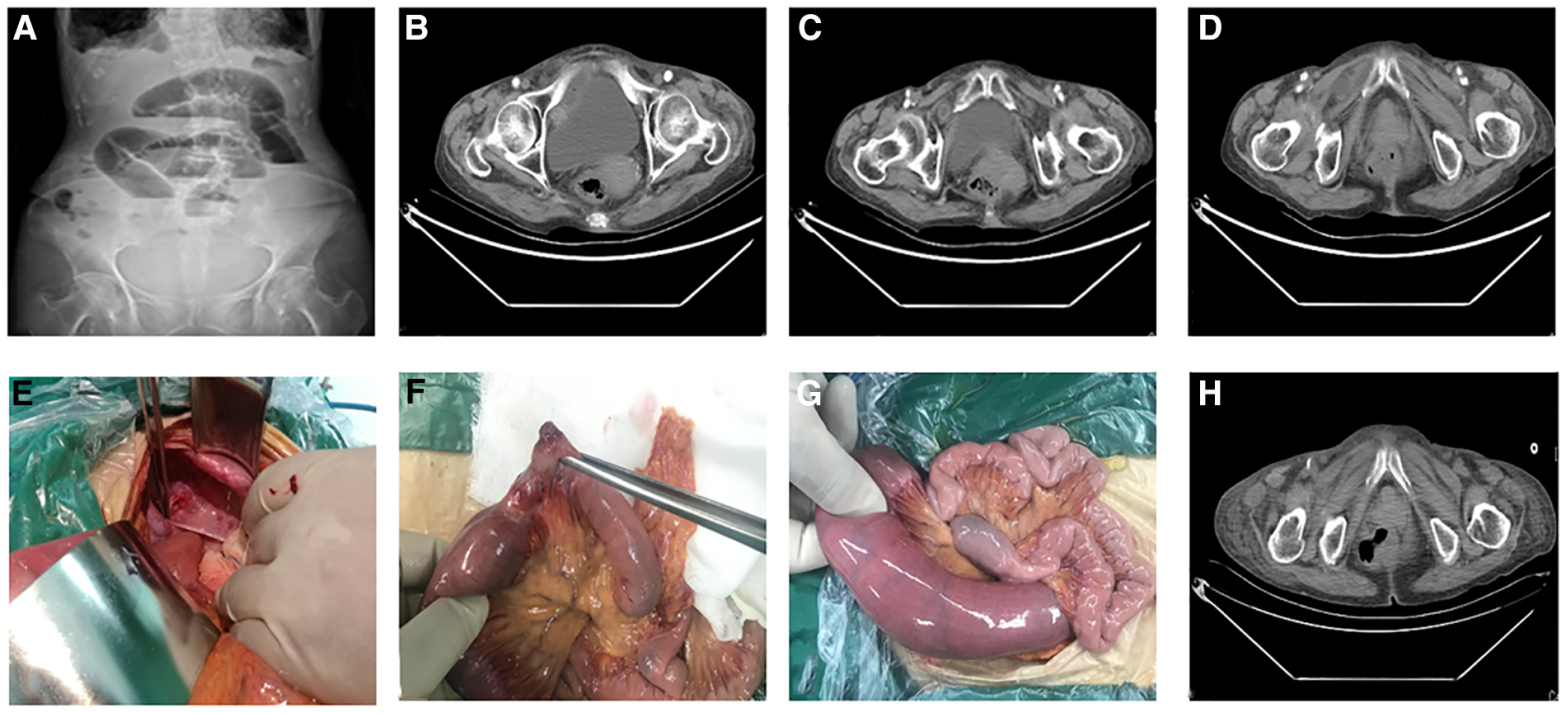
(**A**) Preoperative vertical radiographs of the abdomen showing the small bowel obstruction; (**B–D**) preoperative pelvic CT showing intestinal hernia in the obturator foramen; (**E–G)** Ischemic necrosis occurred in part of the small intestine and herniated into the obturator foramen during the operation; (**H**) Pelvic CT reexamination showed that the obturator foramen had returned to normal.

The patient was an 86-year-old woman admitted to hospital because of abdominal pain and abdominal distension with constipation for 5 days. The patient developed abdominal pain and flatulence after eating 5 days previously, accompanied by nausea, vomiting (once) of food and bile, and constipation, without acid reflux, belching, frequent urination, urgency, dysuria, shivering or fever. However, the abdominal distension persisted, so the patient went to the local hospital. After an abdominal computed tomography (CT) scan and various other examinations, intestinal obstruction and double lung inflammation were considered, and she was treated with gastrointestinal decompression, somatostatin (to inhibit the secretion of digestive fluid), and rehydration (for which the specific medication and dosage are unknown) for 4 days. However, the abdominal distension did not improve significantly, so she was transferred to our hospital for treatment. The abdominal standing plain x-ray film showed gas shadows and the gas-fluid levels in the intestine, but there was no free gas under the diaphragm (Figure 1A). The patient was admitted to the outpatient department of our hospital with an “intestinal obstruction”. The patient had not defecated since the onset of the disease but urinated normally. She had a history of hypertension for more than 30 years, long-term oral antihypertensive drug use, and acceptably controlled blood pressure. She also had a history of constipation for >10 years. Physical examination showed weight loss, abdominal distension, and tenderness of the entire abdomen.

When the patient is instructed to straighten the legs and rotate them outward, pain occurs in the right inner thigh with a sense of soreness.

### 2.2. Imaging findings

The patient's condition improved after admission. Routine blood examination revealed a leukocyte count of 10.6 × 10^9^/L; no other results were abnormal. CT enhancement of the entire abdomen and pelvis showed that the intestines in the left abdomen and pelvic cavity areas were “aggregated”. The small intestinal wall on the right side of the pelvic cavity was thickened, with mild and uneven enhancement. Some of the intestines in the upper segment were dilated, while others showed an accumulation of gas and effusion, and some were flat. The sigmoid colon was long. Dense shadows were seen in the abdominal and pelvic cavities. A convex shadow was seen in the right inguinal area of the intestinal canal, and the corresponding intestinal wall was thickened. This finding was consistent with a small intestinal obstruction or OH (Figures 1B–D).

### 2.3. Therapeutic intervention and histopathological findings

Emergency surgery was performed. A large volume of light yellow ascites was found in the abdominal cavity during exploration. The ascites (700 ml) was absorbed and further exploration showed that the ileal wall was incarcerated at the right obturator 70 cm from the ileocecum. Moreover, the proximal intestine was dilated, the intestinal wall was congested and edematous, the distal intestine was empty, and there were no obvious abnormalities in the intestinal wall. When the incarcerated intestinal wall was returned to its original position, a 3-cm-diameter section of ischemic necrotic tissue was detected (Figures 1E–G). Continuous exploration revealed no obvious abnormalities in the liver, gallbladder, stomach, other parts of the small intestine, colon, or rectum. End-to-end anastomosis of the small intestine was performed after partial resection. Postoperative pathology showed that the whole of the intestinal wall, which is part of the ileum, was congested and bleeding. Furthermore, a local ulcer had formed, granulation tissue exhibited proliferation, a large amount of chronic inflammatory cells infiltrated the surrounding mucosal layer, and lymphoid tissue proliferated in the submucosa.

### 2.4. Follow-up and outcomes

Antibiotic therapy and rehydration were provided after the operation, and no obvious abnormalities were detected by postoperative CT (Figure 1H). The patient was discharged after a successful recovery. No abnormalities were found during the 1- or 3-month follow-up visits.

## 3. Discussion

**Figure 2:**
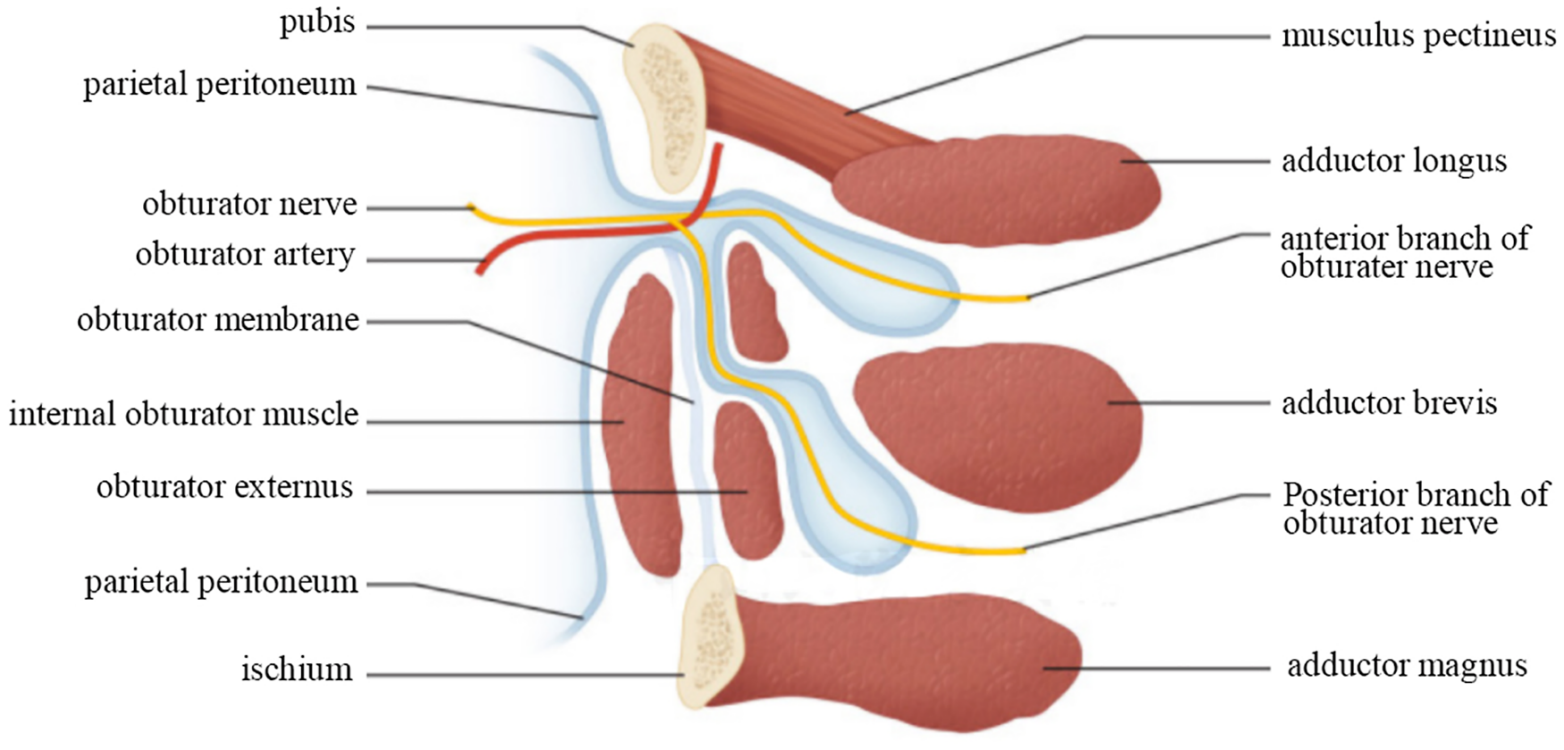
Obturator canal anatomy.

The obturator foramen, located on the anterolateral side of the pelvic wall, directly below the acetabulum, is the largest foramen in the human body. At embryonic week 8, the hip osteondral primordium is obvious, forming an incomplete obturator foramen, obturator membrane, and nerve vascular bundle (4). The ischial ramus rises directly in the middle and merges with the descending pubic ramus to form the obturator foramen. Ossification of the hip bone begins after birth, but the obturator foramen and its membrane are never fully ossified, making it a true foramen. It is generally oval in shape and is essentially covered by the fibrous bone obturator membrane, retaining only a small area of the anterior superior part where obturator nerves, arteries, and veins pass through and enter the obturator canal (5). The obturator canal (Figure 2), about 2–3 cm long and 1 cm wide, arises from the defect of the obturator membrane, diagonally downward, and ends in the obturator area of the thigh. The wall of the obturator groove forms the superior and lateral wall of the pubic bone, and the free edge of the obturator membrane forms the inferior wall with the internal and external obturator muscles. The obturator nerve enters the obturator canal above the accompanying arteries and veins, and passes through the obturator canal. It was divided into anterior and posterior segments to innervate the adductor muscle group and the ipsilateral hip and knee joints, respectively (6). Preperitoneal fat and lymphoid tissue (fat bodies) form a cushion in the canal around the obturator neurovascular bundle, which can prevent herniation of abdominal contents. Obturator hernia is highly prevalent in women due to their wider pelvis, larger triangular obturator and larger transverse diameter. A hernia forms easily when the pelvic floor muscles and fascia are weak and relaxed. Older, frail, and emaciated women who have undergone multiple pregnancies and childbirth may develop OH, which has thus been called “little old lady hernia” (7). Conditions that can increase intraabdominal pressure can also cause OH, such as chronic obstructive pulmonary disease, long-term constipation, massive ascites, or kyphosis (8). Because the left side is covered by the sigmoid colon, the incidence ratio between the left and right sides is 1:2; it is rare on both sides (9). Our patient was a thin, elderly woman with a long history of constipation and several children, which are major risk factors for OH.

Because of the deep location, small hernia ring, and difficult-to-reach surface, the clinical manifestations of OH are not specific, and the misdiagnosis rate is high before the operation: the incidence of OH is low, such that it is rarely encountered; the patients are elderly women with other diseases, poor general condition, a high pain threshold, atypical symptoms and signs, and symptoms that are easily masked; and the patient's family may prefer conservative treatment and visit other departments, such as gastroenterology and pain departments. Diffuse peritonitis caused by intestinal perforation and intestinal necrosis, as well as septic shock, may develop. The incidence and mortality rates of septic shock are both 70% (10). Our patient stated that she had experienced abdominal discomfort in the past, which was considered as dyspepsia and not systematically diagnosed or treated.

The clinical manifestations of OH are abdominal pain caused by intestinal obstruction and obturator neuralgia (Howship-Romberg sign) caused by compression of the obturator nerve. More than 70% of patients have an acute intestinal obstruction of unknown etiology. In the initial stage, due to the shallow bottom of the hernia sac, the hernia content is easy to “reset,” and recurrent abdominal pain may develop (11). When an incarcerated bowel cannot be retracted by itself, the patient will show symptoms of acute mechanical intestinal obstruction including nausea and vomiting, abdominal pain, and abdominal distension. When ischemic necrosis occurs late in the case of an incarcerated bowel, the patient will develop acute diffuse peritonitis and systemic septic shock. Some patients suffer from radiating pain due to compression of the obturator nerve above the obturator canal by the protruding hernia sac; this is the Howship-Romberg sign, which is characterized by tingling and numbness of the affected side, radiating from the groin area and the inner thigh to the popliteal fossa, accompanied by paresthesia, cough, and other factors that increase abdominal pressure, hip flexion, external rotation, and thigh extension or abduction; in turn, this exacerbates the pain, and the incidence is 15%–50% (12). The adductor reflex of the affected thigh disappears in some patients, suggesting obturator nerve compression (Hannington-Kiff sign), which is more specific and has a lower incidence. The main clinical manifestation of our patient was intestinal obstruction. The Howship-Romberg and Hannington-Kiff signs were not observed on physical examination. Many factors lead to intestinal obstruction, which undoubtedly increases the difficulty of the diagnosis.

CT is the first choice imaging modality for preoperative diagnosis of OH, given its diagnostic accuracy and specificity of 78%–100% (13). A circular, teardrop-like, or short tubular hernia sac may be seen on CT scans. When the intestinal canal is herniated into the obturator tube, it abruptly narrows in the obturator muscle and internal muscle, and imaging may show a typical “beak-like” change. Along with small intestinal obstruction, the typical CT imaging manifestations of OH include a small intestinal obstruction, bird's beak sign in the obstructive area, and close relationship between the hernia sac and small intestine. Magnetic resonance imaging can also be used to diagnose OH, but is expensive and has less clinical applicability. In addition, ultrasonic diagnosis is more difficult due to interference from intestinal gas. Therefore, abdominal CT is the first choice for patients with an intestinal obstruction.

OH should be treated as early as possible after diagnosis, to reduce the likelihood of complications such as intestinal necrosis, intestinal perforation, peritonitis, and septic shock. The main surgical methods are laparotomy and laparoscopy. Exploratory laparotomy causes trauma, and patients are predominantly females with various diseases. Postoperative complications, such as poor wound healing and pulmonary infection, easily occur. Endoscopic surgery has the advantages of less trauma and postoperative pain, and rapid recovery of digestive tract function. Many studies have focused on the application of endoscopic surgery for OH (14). However, because most patients with OH have an acute intestinal obstruction, perforation, necrosis, or peritonitis, emergency laparotomy is still the main treatment for OH.

A lower abdominal exploratory incision is the preferred surgical approach, because this allows for comprehensive and systematic abdominal exploration. An exploratory laparotomy allows for deep exploration to determine whether there is an occult hernia, femoral or inguinal hernia, and/or small intestinal diverticulum (15). The operator has a clear view and thus can avoid injuring the intestines and bladder, as well as blood vessels and nerves (particularly those that are easily exposed). Fatal bleeding caused by injury to an abnormal blood vessel during the operation can also be avoided. The intestinal resection rate of OH is 25%–50%. When necrosis or perforation occurs during small intestinal incarceration, it must be repaired, resected, and anastomosed. Due to edema and expansion caused by intestinal obstruction, manual anastomosis is safer than endoscopic anastomosis (16). Patients are often complicated with diffuse peritonitis, which responds to peritoneal flushing after the operation. Methods to repair OH defects include simple suturing close to the defect and placement of an artificial patch (17). Regarding suture treatment for OH in our case, the hernia sac was freed and turned over after the contents were “reset”. The sac was then ligated and removed, the mouth of the obturator tube was exposed, and the obturator muscle and obturator fascia were sutured using purse or zigzag sutures to close the obturator. Whether mesh is needed to repair an OH is not clearly stated in current guidelines. After our patient was diagnosed by CT, the median abdominal incision was removed (during the emergency operation). After the 3 cm necrotic area of the bowel had been removed, the right-sided hernia was sutured and closed. The patient was treated with antibiotics and rehydration postoperatively, along with symptomatic support, and discharged after treatment. The postoperative follow-up was uneventful; no obvious abnormalities were found by CT, and there was no complaint of abdominal pain, abdominal distension, or other forms of discomfort after eating.

## 4. Conclusion

Obturator hernia is very rare and difficult to diagnose. When elderly women with long-term chronic diseases, a thin body, or a history of multiple deliveries have the following conditions: (1). Symptoms of intestinal obstruction (2). Sudden abdominal pain due to increased intra-abdominal pressure (3). Pain in the inner thigh and mild abdominal distention only. All patients should be alert to OH. Howship-Romberg sign should be checked during physical examination. If there is a palpable mass in the inguinal region, it should be differentiated from indirect inguinal hernia and direct inguinal hernia. Abdominal CT is the first choice for imaging examination. Once diagnosed, early surgery should be performed to relieve obstruction and repair defects (17). Timely diagnosis and treatment can reduce the occurrence of intestinal necrosis, perforation, sepsis and other adverse events, thereby improving the prognosis of patients.

## Data Availability

The raw data supporting the conclusions of this article will be made available by the authors, without undue reservation.
